# Supplementary material for: Neuronal timescales are functionally dynamic and shaped by cortical microarchitecture
Source: eLife. 2020 Nov 23;9:e61277. doi: 10.7554/eLife.61277 (PMC7755395; doi:10.7554/eLife.61277)
Supplement: Supplementary file 1. [file elife-61277-supp1.docx]

Supplementary File 1**. Significant items from brain-specific GOEA (data for Figure 3F)**

| **gene association** | **ID** | **e/p** | **ontology** | **name** | **enrichment**  **ratio** | **p-value**  **(FDR-adjusted)** |
| --- | --- | --- | --- | --- | --- | --- |
| all | GO:0034702 | e | CC | ion channel complex | 1.959 | 0.008 |
| all | GO:1902495 | e | CC | transmembrane transporter complex | 1.91 | 0.008 |
| all | GO:1990351 | e | CC | transporter complex | 1.91 | 0.008 |
| all | GO:0098982 | e | CC | GABA-ergic synapse | 2.497 | 0.038 |
| all | GO:1902711 | e | CC | GABA-A receptor complex | 4.541 | 0.038 |
| all | GO:0034707 | e | CC | chloride channel complex | 3.385 | 0.038 |
| pos | GO:0008195 | e | MF | phosphatidate phosphatase activity | 13.864 | 0.007 |
| neg | GO:0098660 | e | BP | inorganic ion transmembrane transport | 2.515 | 0.002 |
| neg | GO:0098662 | e | BP | inorganic cation transmembrane transport | 2.529 | 0.007 |
| neg | GO:0098655 | e | BP | cation transmembrane transport | 2.439 | 0.007 |
| neg | GO:0034220 | e | BP | ion transmembrane transport | 2.057 | 0.03 |
| neg | GO:0030001 | e | BP | metal ion transport | 2.239 | 0.036 |
| neg | GO:0071805 | e | BP | potassium ion transmembrane transport | 3.122 | 0.036 |
| neg | GO:0006813 | e | BP | potassium ion transport | 3.081 | 0.037 |
| neg | GO:1902495 | e | CC | transmembrane transporter complex | 2.334 | 0.009 |
| neg | GO:1990351 | e | CC | transporter complex | 2.334 | 0.009 |
| neg | GO:0034702 | e | CC | ion channel complex | 2.36 | 0.009 |
| neg | GO:0098796 | e | CC | membrane protein complex | 2.063 | 0.009 |
| neg | GO:0034703 | e | CC | cation channel complex | 2.379 | 0.03 |
| neg | GO:0005244 | e | MF | voltage-gated ion channel activity | 3.081 | 0.002 |
| neg | GO:0022832 | e | MF | voltage-gated channel activity | 3.081 | 0.002 |
| neg | GO:0046873 | e | MF | metal ion transmembrane transporter activity | 2.453 | 0.002 |
| neg | GO:0022890 | e | MF | inorganic cation transmembrane transporter activity | 2.24 | 0.005 |
| neg | GO:0005216 | e | MF | ion channel activity | 2.289 | 0.006 |
| neg | GO:0008324 | e | MF | cation transmembrane transporter activity | 2.173 | 0.006 |
| neg | GO:0015318 | e | MF | inorganic molecular entity transmembrane transporter activity | 2.04 | 0.006 |
| neg | GO:0015077 | e | MF | monovalent inorganic cation transmembrane transporter activity | 2.535 | 0.006 |
| neg | GO:0015075 | e | MF | ion transmembrane transporter activity | 2.024 | 0.006 |
| neg | GO:0015079 | e | MF | potassium ion transmembrane transporter activity | 3.041 | 0.006 |
| neg | GO:0005215 | e | MF | transporter activity | 1.883 | 0.006 |
| neg | GO:0022857 | e | MF | transmembrane transporter activity | 1.906 | 0.006 |
| neg | GO:0022836 | e | MF | gated channel activity | 2.301 | 0.006 |
| neg | GO:0015267 | e | MF | channel activity | 2.191 | 0.006 |
| neg | GO:0022803 | e | MF | passive transmembrane transporter activity | 2.191 | 0.006 |
| neg | GO:0005249 | e | MF | voltage-gated potassium channel activity | 3.658 | 0.006 |
| neg | GO:0005261 | e | MF | cation channel activity | 2.353 | 0.009 |
| neg | GO:0005267 | e | MF | potassium channel activity | 3.058 | 0.011 |
| neg | GO:0022843 | e | MF | voltage-gated cation channel activity | 2.744 | 0.022 |

e/p: enriched or purified; BP: biological process; CC: cellular components; MF: molecular function
