# Supplementary material for: Neuronal timescales are functionally dynamic and shaped by cortical microarchitecture
Source: eLife. 2020 Nov 23;9:e61277. doi: 10.7554/eLife.61277 (PMC7755395; doi:10.7554/eLife.61277)
Supplement: Supplementary file 2. [file elife-61277-supp2.docx]

Supplementary File 2. **Significant items from all-gene GOEA**

| **gene association** | **ID** | **e/p** | **ontology** | **name** | **enrichment**  **ratio** | **p-value**  **(FDR-adjusted)** |
| --- | --- | --- | --- | --- | --- | --- |
| all | GO:0034702 | e | CC | ion channel complex | 1.83 | 0.008 |
| all | GO:1990351 | e | CC | transporter complex | 1.774 | 0.008 |
| all | GO:1902495 | e | CC | transmembrane transporter complex | 1.79 | 0.008 |
| all | GO:0034703 | e | CC | cation channel complex | 1.952 | 0.009 |
| all | GO:0098982 | e | CC | GABA-ergic synapse | 2.468 | 0.048 |
| all | GO:1902711 | e | CC | GABA-A receptor complex | 5.035 | 0.048 |
| pos | GO:0050866 | e | BP | negative regulation of cell activation | 3.596 | 0 |
| pos | GO:0002376 | e | BP | immune system process | 1.629 | 0 |
| pos | GO:0006955 | e | BP | immune response | 1.992 | 0 |
| pos | GO:0002695 | e | BP | negative regulation of leukocyte activation | 3.343 | 0.001 |
| pos | GO:0045087 | e | BP | innate immune response | 2.297 | 0.005 |
| pos | GO:0050865 | e | BP | regulation of cell activation | 2.099 | 0.005 |
| pos | GO:0045321 | e | BP | leukocyte activation | 1.834 | 0.006 |
| pos | GO:0007165 | e | BP | signal transduction | 1.301 | 0.006 |
| pos | GO:0051250 | e | BP | negative regulation of lymphocyte activation | 3.305 | 0.007 |
| pos | GO:0070663 | e | BP | regulation of leukocyte proliferation | 2.82 | 0.007 |
| pos | GO:0002252 | e | BP | immune effector process | 1.778 | 0.009 |
| pos | GO:0050670 | e | BP | regulation of lymphocyte proliferation | 2.823 | 0.009 |
| pos | GO:0032944 | e | BP | regulation of mononuclear cell proliferation | 2.807 | 0.009 |
| pos | GO:0050776 | e | BP | regulation of immune response | 1.787 | 0.011 |
| pos | GO:0002682 | e | BP | regulation of immune system process | 1.571 | 0.015 |
| pos | GO:0046634 | e | BP | regulation of alpha-beta T cell activation | 3.772 | 0.016 |
| pos | GO:0001775 | e | BP | cell activation | 1.709 | 0.016 |
| pos | GO:0032956 | e | BP | regulation of actin cytoskeleton organization | 2.229 | 0.016 |
| pos | GO:0003150 | e | BP | muscular septum morphogenesis | 17.672 | 0.016 |
| pos | GO:0032945 | e | BP | negative regulation of mononuclear cell proliferation | 4.208 | 0.016 |
| pos | GO:0050672 | e | BP | negative regulation of lymphocyte proliferation | 4.208 | 0.016 |
| pos | GO:0006952 | e | BP | defense response | 1.686 | 0.016 |
| pos | GO:0002694 | e | BP | regulation of leukocyte activation | 2.013 | 0.016 |
| pos | GO:0002253 | e | BP | activation of immune response | 2.183 | 0.016 |
| pos | GO:0030833 | e | BP | regulation of actin filament polymerization | 2.832 | 0.016 |
| pos | GO:0032970 | e | BP | regulation of actin filament-based process | 2.136 | 0.017 |
| pos | GO:0002684 | e | BP | positive regulation of immune system process | 1.708 | 0.017 |
| pos | GO:0046640 | e | BP | regulation of alpha-beta T cell proliferation | 6.094 | 0.017 |
| pos | GO:0050868 | e | BP | negative regulation of T cell activation | 3.381 | 0.017 |
| pos | GO:0002274 | e | BP | myeloid leukocyte activation | 1.926 | 0.017 |
| pos | GO:0008064 | e | BP | regulation of actin polymerization or depolymerization | 2.697 | 0.017 |
| pos | GO:0030832 | e | BP | regulation of actin filament length | 2.681 | 0.017 |
| pos | GO:0006334 | e | BP | nucleosome assembly | 3.053 | 0.018 |
| pos | GO:0070664 | e | BP | negative regulation of leukocyte proliferation | 3.956 | 0.018 |
| pos | GO:0038096 | e | BP | Fc-gamma receptor signaling pathway involved in phagocytosis | 3.787 | 0.026 |
| pos | GO:0002433 | e | BP | immune response-regulating cell surface receptor signaling pathway involved in phagocytosis | 3.787 | 0.026 |
| pos | GO:0098883 | e | BP | synapse pruning | 10.041 | 0.027 |
| pos | GO:0038094 | e | BP | Fc-gamma receptor signaling pathway | 3.734 | 0.029 |
| pos | GO:0051249 | e | BP | regulation of lymphocyte activation | 2.035 | 0.029 |
| pos | GO:0002431 | e | BP | Fc receptor mediated stimulatory signaling pathway | 3.682 | 0.03 |
| pos | GO:0042116 | e | BP | macrophage activation | 4.734 | 0.03 |
| pos | GO:0110053 | e | BP | regulation of actin filament organization | 2.279 | 0.03 |
| pos | GO:0150064 | e | BP | vertebrate eye-specific patterning | 22.09 | 0.03 |
| pos | GO:0002683 | e | BP | negative regulation of immune system process | 2.008 | 0.03 |
| pos | GO:0051049 | e | BP | regulation of transport | 1.428 | 0.03 |
| pos | GO:0098542 | e | BP | defense response to other organism | 1.811 | 0.033 |
| pos | GO:0150146 | e | BP | cell junction disassembly | 9.204 | 0.033 |
| pos | GO:0016322 | e | BP | neuron remodeling | 9.204 | 0.033 |
| pos | GO:1903038 | e | BP | negative regulation of leukocyte cell-cell adhesion | 3.04 | 0.033 |
| pos | GO:0007166 | e | BP | cell surface receptor signaling pathway | 1.391 | 0.034 |
| pos | GO:0034728 | e | BP | nucleosome organization | 2.591 | 0.037 |
| pos | GO:0036336 | e | BP | dendritic cell migration | 6.976 | 0.037 |
| pos | GO:0048584 | e | BP | positive regulation of response to stimulus | 1.379 | 0.039 |
| pos | GO:0001774 | e | BP | microglial cell activation | 5.727 | 0.039 |
| pos | GO:0002269 | e | BP | leukocyte activation involved in inflammatory response | 5.727 | 0.039 |
| pos | GO:0050778 | e | BP | positive regulation of immune response | 1.82 | 0.039 |
| pos | GO:2000112 | p | BP | regulation of cellular macromolecule biosynthetic process | 0.718 | 0.017 |
| pos | GO:0051252 | p | BP | regulation of RNA metabolic process | 0.718 | 0.019 |
| pos | GO:0044271 | p | BP | cellular nitrogen compound biosynthetic process | 0.554 | 0.029 |
| pos | GO:0019219 | p | BP | regulation of nucleobase-containing compound metabolic process | 0.737 | 0.029 |
| pos | GO:0090304 | p | BP | nucleic acid metabolic process | 0.654 | 0.037 |
| pos | GO:0032993 | e | CC | protein-DNA complex | 2.829 | 0.016 |
| pos | GO:0000786 | e | CC | nucleosome | 3.488 | 0.016 |
| pos | GO:0005887 | e | CC | integral component of plasma membrane | 1.555 | 0.016 |
| pos | GO:0031226 | e | CC | intrinsic component of plasma membrane | 1.536 | 0.016 |
| pos | GO:0044815 | e | CC | DNA packaging complex | 3.217 | 0.023 |
| pos | GO:0030666 | e | CC | endocytic vesicle membrane | 2.705 | 0.034 |
| pos | GO:0031514 | e | CC | motile cilium | 2.897 | 0.034 |
| pos | GO:0043235 | e | CC | receptor complex | 1.981 | 0.04 |
| pos | GO:0000839 | e | CC | Hrd1p ubiquitin ligase ERAD-L complex | 11.045 | 0.047 |
| pos | GO:0016021 | e | CC | integral component of membrane | 1.232 | 0.047 |
| pos | GO:0005634 | p | CC | nucleus | 0.79 | 0.04 |
| pos | GO:0003676 | p | MF | nucleic acid binding | 0.694 | 0.012 |
| neg | GO:0006813 | e | BP | potassium ion transport | 2.911 | 0.004 |
| neg | GO:0071805 | e | BP | potassium ion transmembrane transport | 2.868 | 0.008 |
| neg | GO:0015079 | e | MF | potassium ion transmembrane transporter activity | 2.888 | 0.001 |
| neg | GO:0015075 | e | MF | ion transmembrane transporter activity | 1.726 | 0.001 |
| neg | GO:0022857 | e | MF | transmembrane transporter activity | 1.649 | 0.001 |
| neg | GO:0046873 | e | MF | metal ion transmembrane transporter activity | 2.068 | 0.001 |
| neg | GO:0005215 | e | MF | transporter activity | 1.587 | 0.002 |
| neg | GO:0022832 | e | MF | voltage-gated channel activity | 2.468 | 0.006 |
| neg | GO:0005244 | e | MF | voltage-gated ion channel activity | 2.468 | 0.006 |
| neg | GO:0015318 | e | MF | inorganic molecular entity transmembrane transporter activity | 1.662 | 0.008 |
| neg | GO:0001227 | e | MF | DNA-binding transcription repressor activity, RNA polymerase II-specific | 2.227 | 0.011 |
| neg | GO:0001217 | e | MF | DNA-binding transcription repressor activity | 2.218 | 0.011 |
| neg | GO:0022836 | e | MF | gated channel activity | 2.007 | 0.015 |
| neg | GO:0005249 | e | MF | voltage-gated potassium channel activity | 3.126 | 0.015 |
| neg | GO:0015077 | e | MF | monovalent inorganic cation transmembrane transporter activity | 1.916 | 0.022 |
| neg | GO:0005267 | e | MF | potassium channel activity | 2.703 | 0.022 |
| neg | GO:0022890 | e | MF | inorganic cation transmembrane transporter activity | 1.701 | 0.033 |

e/p: enriched or purified; BP: biological process; CC: cellular components; MF: molecular function
