# Supplementary material for: Neuronal timescales are functionally dynamic and shaped by cortical microarchitecture
Source: eLife. 2020 Nov 23;9:e61277. doi: 10.7554/eLife.61277 (PMC7755395; doi:10.7554/eLife.61277)
Supplement: Supplementary file 3. [file elife-61277-supp3.docx]

Supplementary File 3. **Significant items from all-gene GOEA with T1w/T2w as control**

| **gene association** | **ID** | **e/p** | **ontology** | **name** | **enrichment**  **ratio** | **p-value**  **(FDR-adjusted)** |
| --- | --- | --- | --- | --- | --- | --- |
| all | GO:1990351 | e | CC | transporter complex | 1.564 | 0.038 |
| all | GO:1902495 | e | CC | transmembrane transporter complex | 1.563 | 0.038 |
| all | GO:0034702 | e | CC | ion channel complex | 1.572 | 0.043 |
| all | GO:1990904 | p | CC | ribonucleoprotein complex | 0.641 | 0.038 |
| pos | GO:0006955 | e | BP | immune response | 1.647 | 0.001 |
| pos | GO:0045087 | e | BP | innate immune response | 1.963 | 0.001 |
| pos | GO:0002274 | e | BP | myeloid leukocyte activation | 1.789 | 0.001 |
| pos | GO:0002376 | e | BP | immune system process | 1.383 | 0.001 |
| pos | GO:0002684 | e | BP | positive regulation of immune system process | 1.607 | 0.001 |
| pos | GO:0050865 | e | BP | regulation of cell activation | 1.778 | 0.002 |
| pos | GO:0045321 | e | BP | leukocyte activation | 1.602 | 0.002 |
| pos | GO:0042116 | e | BP | macrophage activation | 4.158 | 0.002 |
| pos | GO:0050776 | e | BP | regulation of immune response | 1.605 | 0.002 |
| pos | GO:0002694 | e | BP | regulation of leukocyte activation | 1.802 | 0.002 |
| pos | GO:0050670 | e | BP | regulation of lymphocyte proliferation | 2.287 | 0.004 |
| pos | GO:0002682 | e | BP | regulation of immune system process | 1.427 | 0.005 |
| pos | GO:0070663 | e | BP | regulation of leukocyte proliferation | 2.256 | 0.005 |
| pos | GO:0002253 | e | BP | activation of immune response | 1.905 | 0.005 |
| pos | GO:0032944 | e | BP | regulation of mononuclear cell proliferation | 2.274 | 0.006 |
| pos | GO:0150146 | e | BP | cell junction disassembly | 7.277 | 0.006 |
| pos | GO:0050778 | e | BP | positive regulation of immune response | 1.697 | 0.006 |
| pos | GO:0006898 | e | BP | receptor-mediated endocytosis | 2.296 | 0.007 |
| pos | GO:0030833 | e | BP | regulation of actin filament polymerization | 2.319 | 0.008 |
| pos | GO:0001775 | e | BP | cell activation | 1.504 | 0.008 |
| pos | GO:0051249 | e | BP | regulation of lymphocyte activation | 1.806 | 0.009 |
| pos | GO:0034314 | e | BP | Arp2/3 complex-mediated actin nucleation | 5.871 | 0.009 |
| pos | GO:0002252 | e | BP | immune effector process | 1.521 | 0.009 |
| pos | GO:0050866 | e | BP | negative regulation of cell activation | 2.248 | 0.009 |
| pos | GO:0001774 | e | BP | microglial cell activation | 4.62 | 0.009 |
| pos | GO:0045010 | e | BP | actin nucleation | 4.62 | 0.009 |
| pos | GO:0002269 | e | BP | leukocyte activation involved in inflammatory response | 4.62 | 0.009 |
| pos | GO:0032940 | e | BP | secretion by cell | 1.49 | 0.011 |
| pos | GO:0045055 | e | BP | regulated exocytosis | 1.598 | 0.012 |
| pos | GO:0140352 | e | BP | export from cell | 1.475 | 0.012 |
| pos | GO:0002697 | e | BP | regulation of immune effector process | 1.787 | 0.019 |
| pos | GO:0032103 | e | BP | positive regulation of response to external stimulus | 1.702 | 0.02 |
| pos | GO:0002366 | e | BP | leukocyte activation involved in immune response | 1.621 | 0.02 |
| pos | GO:0006887 | e | BP | exocytosis | 1.539 | 0.021 |
| pos | GO:0098754 | e | BP | detoxification | 2.45 | 0.021 |
| pos | GO:0051046 | e | BP | regulation of secretion | 1.505 | 0.024 |
| pos | GO:0050863 | e | BP | regulation of T cell activation | 1.865 | 0.025 |
| pos | GO:0098883 | e | BP | synapse pruning | 6.805 | 0.025 |
| pos | GO:1903530 | e | BP | regulation of secretion by cell | 1.521 | 0.025 |
| pos | GO:0002263 | e | BP | cell activation involved in immune response | 1.609 | 0.026 |
| pos | GO:0048584 | e | BP | positive regulation of response to stimulus | 1.284 | 0.027 |
| pos | GO:0043299 | e | BP | leukocyte degranulation | 1.656 | 0.027 |
| pos | GO:0038096 | e | BP | Fc-gamma receptor signaling pathway involved in phagocytosis | 2.851 | 0.027 |
| pos | GO:0002433 | e | BP | immune response-regulating cell surface receptor signaling pathway involved in phagocytosis | 2.851 | 0.027 |
| pos | GO:0002695 | e | BP | negative regulation of leukocyte activation | 2.216 | 0.029 |
| pos | GO:0016192 | e | BP | vesicle-mediated transport | 1.33 | 0.03 |
| pos | GO:0002275 | e | BP | myeloid cell activation involved in immune response | 1.64 | 0.03 |
| pos | GO:0038094 | e | BP | Fc-gamma receptor signaling pathway | 2.811 | 0.03 |
| pos | GO:0002696 | e | BP | positive regulation of leukocyte activation | 1.84 | 0.031 |
| pos | GO:0032722 | e | BP | positive regulation of chemokine production | 3.18 | 0.033 |
| pos | GO:0002431 | e | BP | Fc receptor mediated stimulatory signaling pathway | 2.772 | 0.033 |
| pos | GO:0009607 | e | BP | response to biotic stimulus | 1.432 | 0.033 |
| pos | GO:0043207 | e | BP | response to external biotic stimulus | 1.435 | 0.035 |
| pos | GO:0016322 | e | BP | neuron remodeling | 6.238 | 0.035 |
| pos | GO:0008064 | e | BP | regulation of actin polymerization or depolymerization | 2.103 | 0.037 |
| pos | GO:0030832 | e | BP | regulation of actin filament length | 2.091 | 0.038 |
| pos | GO:1903037 | e | BP | regulation of leukocyte cell-cell adhesion | 1.821 | 0.04 |
| pos | GO:0032930 | e | BP | positive regulation of superoxide anion generation | 5.137 | 0.041 |
| pos | GO:0042129 | e | BP | regulation of T cell proliferation | 2.202 | 0.042 |
| pos | GO:0055069 | e | BP | zinc ion homeostasis | 4.01 | 0.045 |
| pos | GO:0042534 | e | BP | regulation of tumor necrosis factor biosynthetic process | 4.01 | 0.045 |
| pos | GO:0061900 | e | BP | glial cell activation | 3.669 | 0.046 |
| pos | GO:0002283 | e | BP | neutrophil activation involved in immune response | 1.627 | 0.046 |
| pos | GO:0042119 | e | BP | neutrophil activation | 1.626 | 0.049 |
| pos | GO:0051252 | p | BP | regulation of RNA metabolic process | 0.704 | 0 |
| pos | GO:2000112 | p | BP | regulation of cellular macromolecule biosynthetic process | 0.711 | 0 |
| pos | GO:2001141 | p | BP | regulation of RNA biosynthetic process | 0.704 | 0 |
| pos | GO:0019219 | p | BP | regulation of nucleobase-containing compound metabolic process | 0.729 | 0 |
| pos | GO:1903506 | p | BP | regulation of nucleic acid-templated transcription | 0.705 | 0 |
| pos | GO:0010556 | p | BP | regulation of macromolecule biosynthetic process | 0.733 | 0 |
| pos | GO:0006355 | p | BP | regulation of transcription, DNA-templated | 0.713 | 0 |
| pos | GO:0010468 | p | BP | regulation of gene expression | 0.77 | 0 |
| pos | GO:0031326 | p | BP | regulation of cellular biosynthetic process | 0.757 | 0.001 |
| pos | GO:0080090 | p | BP | regulation of primary metabolic process | 0.812 | 0.001 |
| pos | GO:0009889 | p | BP | regulation of biosynthetic process | 0.769 | 0.001 |
| pos | GO:0060255 | p | BP | regulation of macromolecule metabolic process | 0.829 | 0.002 |
| pos | GO:0051171 | p | BP | regulation of nitrogen compound metabolic process | 0.819 | 0.002 |
| pos | GO:0019222 | p | BP | regulation of metabolic process | 0.846 | 0.005 |
| pos | GO:0031323 | p | BP | regulation of cellular metabolic process | 0.837 | 0.006 |
| pos | GO:0090304 | p | BP | nucleic acid metabolic process | 0.739 | 0.029 |
| pos | GO:0005887 | e | CC | integral component of plasma membrane | 1.417 | 0.016 |
| pos | GO:0031226 | e | CC | intrinsic component of plasma membrane | 1.396 | 0.016 |
| pos | GO:0005885 | e | CC | Arp2/3 protein complex | 7.485 | 0.017 |
| pos | GO:0030666 | e | CC | endocytic vesicle membrane | 2.291 | 0.017 |
| pos | GO:0070062 | e | CC | extracellular exosome | 1.285 | 0.042 |
| pos | GO:1903561 | e | CC | extracellular vesicle | 1.279 | 0.042 |
| pos | GO:0043230 | e | CC | extracellular organelle | 1.278 | 0.042 |
| pos | GO:0016021 | e | CC | integral component of membrane | 1.173 | 0.042 |
| pos | GO:0031224 | e | CC | intrinsic component of membrane | 1.165 | 0.043 |
| pos | GO:0098797 | e | CC | plasma membrane protein complex | 1.559 | 0.043 |
| pos | GO:0005634 | p | CC | nucleus | 0.803 | 0.004 |
| pos | GO:0043229 | p | CC | intracellular organelle | 0.905 | 0.042 |
| pos | GO:0043231 | p | CC | intracellular membrane-bounded organelle | 0.891 | 0.043 |
| pos | GO:0000785 | p | CC | chromatin | 0.633 | 0.043 |
| pos | GO:0003676 | p | MF | nucleic acid binding | 0.69 | 0 |
| pos | GO:0097159 | p | MF | organic cyclic compound binding | 0.78 | 0 |
| pos | GO:1901363 | p | MF | heterocyclic compound binding | 0.779 | 0 |
| pos | GO:0003677 | p | MF | DNA binding | 0.687 | 0.001 |
| pos | GO:0140110 | p | MF | transcription regulator activity | 0.636 | 0.004 |
| pos | GO:0003700 | p | MF | DNA-binding transcription factor activity | 0.592 | 0.008 |
| neg | GO:0034220 | e | BP | ion transmembrane transport | 1.619 | 0.013 |
| neg | GO:0006820 | e | BP | anion transport | 1.785 | 0.013 |
| neg | GO:0006811 | e | BP | ion transport | 1.486 | 0.013 |
| neg | GO:0055085 | e | BP | transmembrane transport | 1.486 | 0.015 |
| neg | GO:0098660 | e | BP | inorganic ion transmembrane transport | 1.674 | 0.035 |
| neg | GO:0098656 | e | BP | anion transmembrane transport | 2.067 | 0.04 |
| neg | GO:0006813 | e | BP | potassium ion transport | 2.359 | 0.046 |
| neg | GO:0015075 | e | MF | ion transmembrane transporter activity | 1.702 | 0 |
| neg | GO:0005215 | e | MF | transporter activity | 1.58 | 0 |
| neg | GO:0022857 | e | MF | transmembrane transporter activity | 1.615 | 0 |
| neg | GO:0046873 | e | MF | metal ion transmembrane transporter activity | 1.978 | 0 |
| neg | GO:0015318 | e | MF | inorganic molecular entity transmembrane transporter activity | 1.67 | 0.001 |
| neg | GO:0008509 | e | MF | anion transmembrane transporter activity | 1.983 | 0.007 |
| neg | GO:0015079 | e | MF | potassium ion transmembrane transporter activity | 2.341 | 0.031 |
| neg | GO:0022890 | e | MF | inorganic cation transmembrane transporter activity | 1.625 | 0.045 |
| neg | GO:0008514 | e | MF | organic anion transmembrane transporter activity | 2.111 | 0.045 |

e/p: enriched or purified; BP: biological process; CC: cellular components; MF: molecular function
